# Vertebral Artery Dissection in a Young Adult: A Case Report

**DOI:** 10.7759/cureus.58100

**Published:** 2024-04-12

**Authors:** Ava Toluie, Anthony T Joseph, Peter A Hrehorovich

**Affiliations:** 1 Pediatrics, Lake Erie College of Osteopathic Medicine, Bradenton, USA; 2 Orthopedic Surgery, Lake Erie College of Osteopathic Medicine, Bradenton, USA; 3 Diagnostic Radiology, Advent Health Sebring, Sebring, USA

**Keywords:** neurology, dissection, cervical spine manipulation, manipulation, infarct, hematoma, pica, posterior inferior cerebellar artery, vad, vertebral artery dissection

## Abstract

Vertebral artery dissections (VAD) pose a significant risk for strokes, particularly in young adults. This case report details the presentation and management of a 48-year-old patient who was diagnosed with an extracranial VAD following cervical spine manipulation (CSM). The patient's symptoms included acute right-sided ataxia, giddiness, vertigo, nausea, vomiting, and persistent pain behind the right ear, prompting immediate evaluation. After ruling out acute intracerebral hemorrhages, a computed tomography angiogram (CTA) of the head and neck identified a severe narrowing of the right distal vertebral artery with a string sign at the level of the right C1 loop (V3 segment), indicating an extracranial VAD. This finding was further supported when ultrasound (US) imaging revealed a high resistance flow pattern in the right distal vertebral artery.

Furthermore, T2 and diffusion-weighted magnetic resonance imaging (MRI) confirmed a 1.8 cm VAD/hematoma and a 1.4 cm acute/subacute infarct in the right posterior inferior cerebellar artery (PICA) territory. This research accentuates the importance of recognizing and addressing that neck pain can be a symptom of musculoskeletal dysfunction or could have neurovascular origins. In this case, the patient's neck pain may have been musculoskeletal or could have been due to a previous dissection. Thus, differentiation should be considered before cervical spine manipulation.

## Introduction

Vertebral artery dissections (VAD), especially extracranial dissections, are a common cause of cerebrovascular accidents (CVA) or strokes in young adults (under age 49) [1-4]. Patients typically present with nonspecific symptoms, including vertigo, dizziness, headaches, occipital pain, and/or neck pain [1]. The significant consequences of VAD include posterior circulation ischemia, ischemic stroke, and transient ischemic attacks [5]. Neurologic deficits of lateral medullary syndrome (LMS) have also been previously reported in patients with VAD [4]. Furthermore, VAD is the most common cause of LMS in younger populations [6]. The etiology of VAD varies as some arise spontaneously while others may occur after minor trauma such as cervical spine manipulations (CSM) [7,8]. This case report highlights a young patient who underwent CSM for neck pain and subsequently developed additional symptoms leading to a diagnosis of an extracranial VAD.

## Case presentation

A 48-year-old patient presented to the emergency department immediately after chiropractic manipulation of the cervical spine with acute right-sided ataxia associated with giddiness, dizziness, vertigo, nausea, vomiting, and constant pain behind the right ear. The patient denied diplopia, dysarthria, and dysphagia. The patient had a past medical history of hyperlipidemia. The patient explained that the reason they sought CSM was that they had been experiencing right-sided neck pain for two weeks, which was a 9/10 in pain and radiated from the right shoulder up.

On a physical exam, the patient was hypertensive (170/90 mmHg) and tachypneic (22 breaths per minute). The patient was oriented to person, place, and time. The patient had no motor or sensory deficits in her upper and lower extremities. Cranial nerve testing of II - XII was intact except for right lateral gaze nystagmus. The patient did not have dysmetria or lateral cerebellar deficits when examined with the finger-to-nose (FNT) and heel-to-shin (HST) tests. Upon ambulation, the patient was ataxic to the right side.

**Figure 1:**
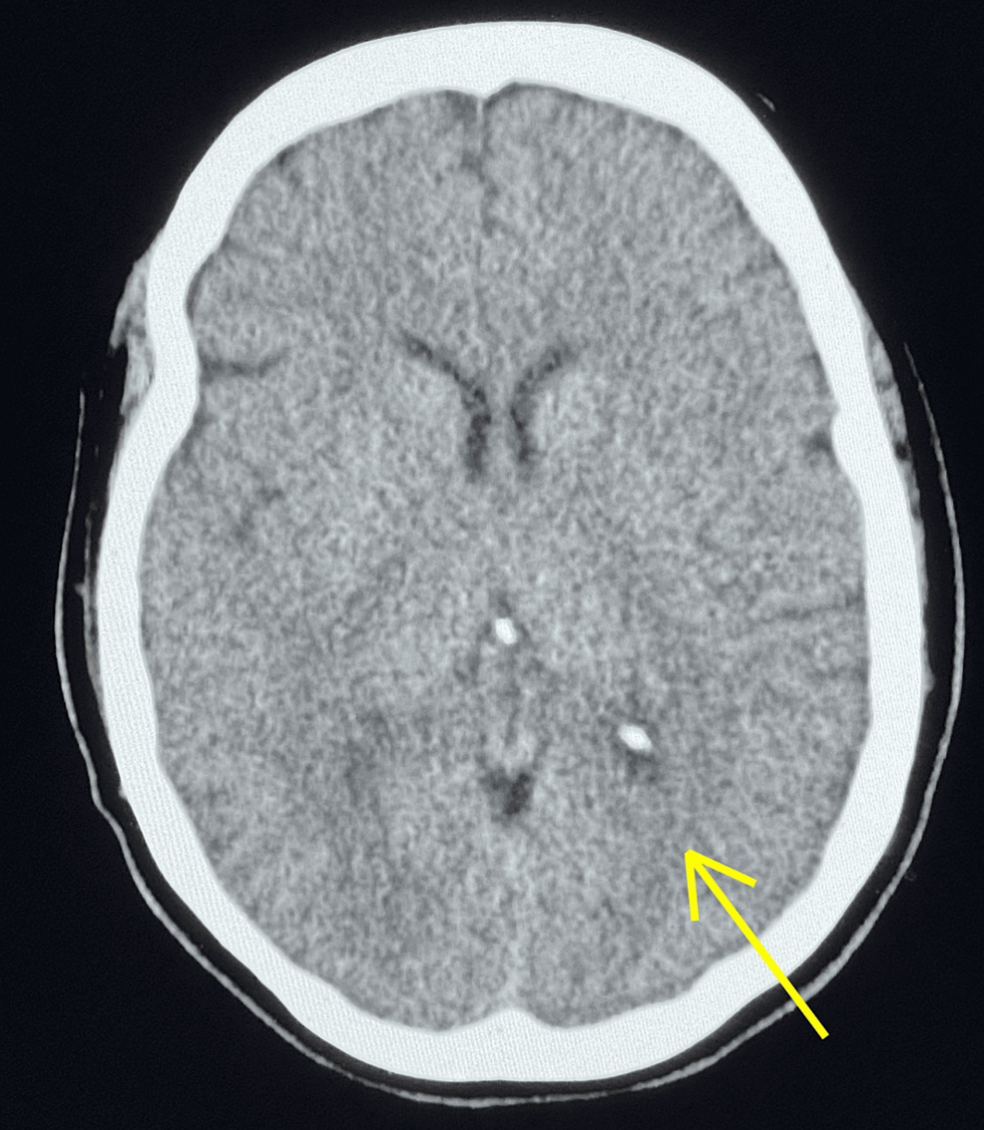
Head CT without contrast, revealing no acute intracranial abnormality CT: computed tomography

The patient was placed on stroke alert protocol, and dual antiplatelet therapy (DAPT) was initiated with aspirin 324 mg and clopidogrel 300 mg. dizziness was managed with meclizine 25 mg as needed. Maintenance dosing for DAPT was continued during the hospital stay. The patient's NIH stroke score (NIHSS) was one (minor severity) due to right lateral nystagmus. The patient was not a candidate for IV tPA or large vessel revascularization. Computed tomography of the head without contrast revealed no intracranial abnormality or hemorrhagic stroke (Figure 1).

**Figure 2:**
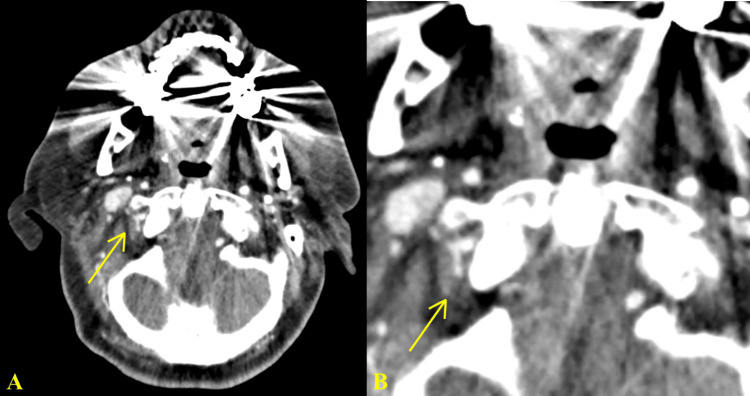
CTA of the neck displaying severe narrowing of the right distal vertebral artery with a string sign at the level of the right C1 loop, most concerning for right distal vertebral artery dissection at this level (A). A magnified image better visualizes the string sign (B). CTA: computed tomography angiogram

Neurology was consulted, resulting in a computed tomography angiogram (CTA) of the neck that revealed extensive narrowing of the right distal vertebral artery, demonstrated by a string sign at the level of the C1 loop in the right vertebral artery (Figure 2). As the right vertebral artery entered the skull, it increased in caliber and was co-dominant with the caliber of the left vertebral artery, indicating no vertebral artery dominance (Figure 2).

**Figure 3:**
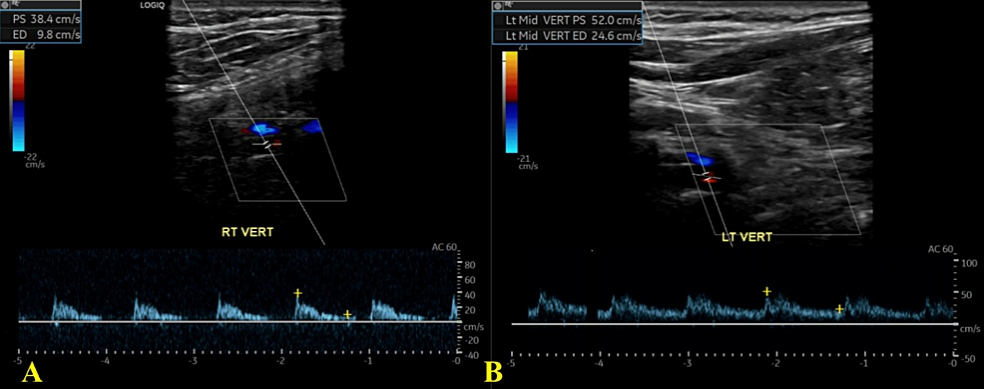
US waveform imaging depicts a high resistance pattern in the right vertebral artery (A) compared to a normal left vertebral artery (B) US: ultrasound

A CTA of the head revealed no abnormalities in the anterior cerebral vasculature. There was, however, moderate focal narrowing of the proximal right P1 segment of the PCA. Ultrasound imaging of the carotid and vertebral arteries revealed a high resistance flow pattern on the spectral anterograde curve. The high resistance flow pattern on ultrasound raises suspicion for a right VAD or, in some cases, indicates the presence of a vertebral artery terminating into the posterior inferior cerebellar artery (PICA) (Figure 3).

**Figure 4:**
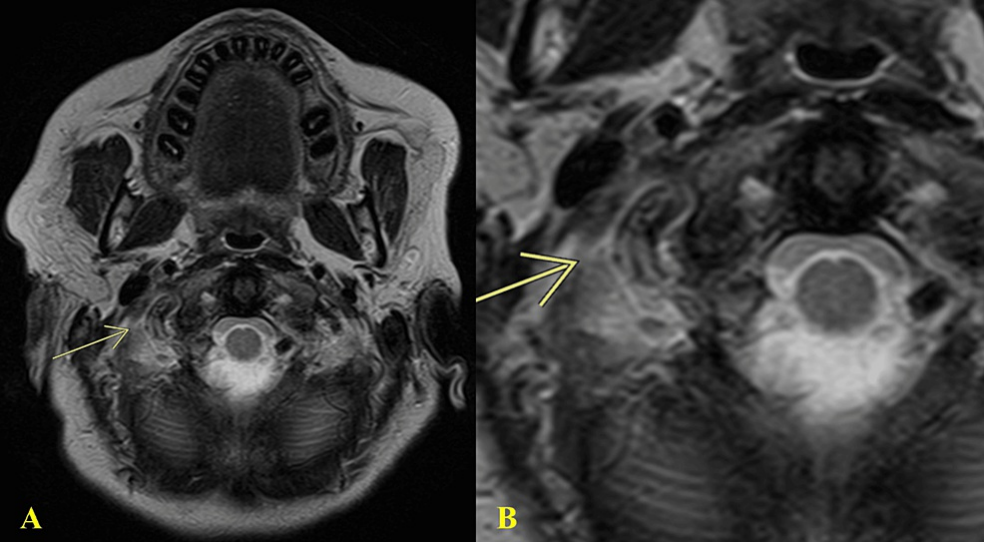
T2 axial MRI displays an impaired flow void of the distal right vertebral artery V3 segment representing known vertebral artery dissection and hematoma, measuring about 1.8 cm in length (A). A magnified image better depicts impaired flow void (B) MRI: magnetic resonance imaging

Magnetic resonance imaging (MRI) without contrast was performed to confirm the diagnosis of a right VAD at the level of the C1 loop. T2 axial imaging showed that the distal right vertebral artery V3 segment had an impaired flow void, confirming the 1.8 cm VAD with a hematoma (Figure 4). There was a normal signal void on the left vertebral artery and an abnormal one on the right, with an increased T2 signal representing hematoma (Figure 4).

**Figure 5:**
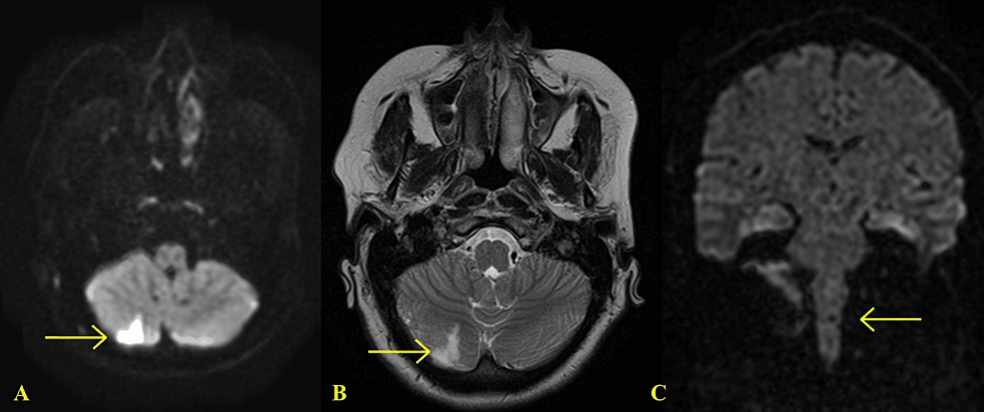
MRI without contrast axial DWI sequence (A) and T2 weighted MR without contrast (B) of the brain reveals an acute/subacute infarct in the right posterior inferior cerebellar distribution of 1.4 cm. Coronal DWI shows no signal abnormality of the medulla oblongata (C). MRI: magnetic resonance imaging DWI: diffusion-weighted imaging

T2-weighted MRI and diffusion-weighted imaging (DWI) without contrast displayed an acute/subacute infarct measuring 1.4 cm in the right posterior inferior cerebellum (PICA distribution) with the absence of acute hemorrhage (Figures 5A-5B). Due to the vascular area affected, the medulla oblongata was also examined on MRI and found to be normal (Figure 5C).

The patient's symptoms of headache, lateral nystagmus, and ataxia had slightly improved throughout the hospitalization. Due to the extracranial nature of the dissection, medical management was initiated with aspirin 81 mg and clopidogrel 75 mg (DAPT) for six months. The patient was advised to avoid any heavy lifting and straining to prevent worsening of the dissection. A follow-up CT angiogram of the brain and neck was scheduled in six months to assess for healing and revascularization.

## Discussion

VAD represents a small proportion of the causes of ischemic stroke in the general population but comprises 10 to 25% of those in middle-aged adults (30-45 years old) [3,9]. VAD can be separated into extracranial vertebral artery dissection (EVAD) and intracranial vertebral artery dissections (IVAD). Risk factors for IVADs include hypertension, excessive alcohol use, and aging [10,11]. On the other hand, a history of trauma has been more frequently associated with EVAD [10]. Patients who suffer from EVAD commonly have symptoms of vertigo, nausea, and neck pain and often present with an ischemic stroke [11]. In addition to risk factors and symptomatology, complications of EVAD and IVAD differ and are associated with intramural hematomas and aneurysm formation, respectively [11]. In patients whose IVAD resulted in a stroke, subarachnoid hemorrhage is an unfortunate sequelae 50% of the time and carries a more severe prognosis [1,5,9]. In this case, the patient presented in an acute setting with symptoms of giddiness, vertigo, nausea, and neck pain following manipulation of the cervical spine, further exemplifying the common presenting symptoms found in a young patient with EVAD.

The location of dissections has been widely analyzed across the four segments of the vertebral artery: V1, V2, V3, and V4. Spontaneous VADs have been preponderantly found in the pars transversaria (V2), or atlas loop (V3), where the vertebral artery curves over the top of the atlas (C1) to enter the skull [5]. At the V3 segment, the vertebral artery is most mobile and, therefore, susceptible to dissection and tearing [9,12]. However, other studies have shown that the V1 segment of the vertebral artery is also susceptible to dissection because it does not course through the transverse foramina and lacks tethering [13]. Similarly, in CSM-induced VADs, the V3 segment is also commonly affected [14]. In this patient's EVAD, the dissection was found at the C1 loop of the V3 segment of the vertebral artery, further demonstrating that increased mobility in the V3 segment increases the risk for VAD in the setting of acute CSM. 

A relationship between the number of treatments and the onset of symptoms has not been found [15]. Some patients experienced symptoms after one manipulation, while others did not report symptoms until after multiple treatments [15]. Patients suffering from acute onset head and/or neck pain may have a dissection already forming that CSM can fully transform into pathology [15]. This case report exemplifies a patient with pre-existing neck pain who developed additional EVAD symptoms after one CSM treatment. This emphasizes the importance of a full workup in patients with neck pain, as this symptom can indicate musculoskeletal or neurovascular issues.

In patients with vertebral artery dissection, stroke prevention is paramount when guiding treatment. Treatment is centered around preventing the formation of a thrombus or thromboembolism from forming over the dissection. Anticoagulation or antiplatelet therapy can be achieved with unfractionated heparin, followed by warfarin or DAPT with aspirin and clopidogrel. Studies have shown that there is no benefit to using antiplatelet over anticoagulant therapy, or vice versa, in patients with carotid or vertebral artery dissection [3,16]. Full recovery has been reported to be achieved in 80% of patients with EVAD who were managed medically [9,17]. However, in patients with IVAD, medical management with anticoagulation is contraindicated as there may be a resultant subarachnoid hemorrhage [9]. Recanalization of the VADs has been reported as high as 64% without revascularization or repair of the dissection [17].

## Conclusions

VAD is a rare but significant cause of ischemic stroke, particularly in middle-aged adults. The case presented underscores the importance of recognizing the common symptoms of EVAD, such as vertigo and cervical spine pain, especially following CSM. Patients with neck pain should consider refraining from receiving cervical spine manipulations until they have had a proper workup done to exclude vascular pathology. While most patients with EVAD can be managed medically without lasting deficits, those with IVAD require careful consideration due to the risk of subarachnoid hemorrhage. This case elucidates the importance of considering EVAD after young patients undergo mechanical stress like CSM, especially when neurological symptoms suddenly arise.
